# Is There Still a French Eating Model? A Taxonomy of Eating Behaviors in Adults Living in the Paris Metropolitan Area in 2010

**DOI:** 10.1371/journal.pone.0119161

**Published:** 2015-03-03

**Authors:** Julien Riou, Thomas Lefèvre, Isabelle Parizot, Anne Lhuissier, Pierre Chauvin

**Affiliations:** 1 Sorbonne Universités, UPMC Univ Paris 06, UMR_S 1136, Pierre Louis Institute of Epidemiology and Public Health, Department of social epidemiology, F-75013 Paris, France; 2 INSERM, UMR_S 1136, Pierre Louis Institute of Epidemiology and Public Health, Department of social epidemiology, F-75013 Paris, France; 3 AP-HP, Hôpital Jean-Verdier, Department of Forensic Medicine, F-93140 Bondy, France; 4 CNRS, UMR 8097, Centre Maurice Halbwachs, Research Team on Social Inequalities, F-75014 Paris, France; 5 INRA, UR1303 ALISS, F-94205 Ivry sur Seine Cedex, France; 6 University of Oxford, Department of Sociology, Manor Road, Oxford OX1 3UQ, United Kingdom; Duke University, UNITED STATES

## Abstract

**Background:**

Meal times in France still represent an important moment in everyday life. The model of three rigorously synchronized meals is still followed by a majority of people, while meal frequencies have flattened in other European or North-American countries. We aimed to examine the “French model” of eating behavior by identifying and characterizing distinct meal patterns.

**Methods:**

Analyses were based on data from the SIRS cohort, a representative survey of the adult population in the Paris area. A clustering algorithm was applied to meal variables (number, time, location, with whom the meal is usually shared and activities associated with meals). Regression models were used to investigate associations between patterns and socio-demographic, social environment and perceived food quality variables.

**Results:**

Five different patterns were identified among 2994 participants. The first three types (prevalence 33%, 17% and 24%) followed a three-meal pattern, with differences in locations and social interactions mainly related to time constraints and age. More marked differences were observed in the remaining two types. In the fourth type (prevalence 13%), individuals ate one or two meals per day, often with an irregular schedule, at home and in front of the television. They frequently were unemployed and had lower income. Breakfast skipping, increased snacking and a low adherence to dietary guidelines suggested that this behavior might have health consequences. In the fifth type (12%), people also ate two meals or less per day, possibly with the same consequences on food quality. However, meals were often taken outside the home, in social settings, and individuals following this pattern were typically active, integrated, young people, suggesting that this pattern might be an adaptation to a modern urban lifestyle.

**Conclusions:**

While a majority of the population still follows the three-meal pattern, our analysis distinguished two other eating patterns associated with specific sociological profiles.

## Introduction

Meal times in France still represent an important moment in everyday life. The three-meal pattern, with breakfast between 7 and 8:30 am, lunch between 12 and 1:30 pm and supper between 7 and 8:30 pm, is still followed by a majority of French people, while meal frequencies have flattened in other European or North-American countries [1–4]. Meals, particularly the breakfast and evening meal, are still often taken at a slow pace, principally at home. Snacking between meals is scarce. Even so, some evolution in meal patterns has been detected, notably a decrease in breakfast frequency, a growing proportion of meals taken alone [3–5], and a simplification of meals in terms of content as shown by Poulain [6].

The three meals a day pattern has been linked to several health benefits, such as a lower prevalence of obesity and a higher consumption of fruits and vegetables [3,7–10]. Nonetheless, some of these associations, particularly with respect to children, have recently been challenged [11]. Meals are also an important occasion for socializing, sharing and consolidation of social ties. The relative preservation of this pattern is not consistent with the idea of destructuration of French eating habits as a consequence of growing globalization and standardization [6,12–14]. Preservation does not necessarily imply plain conservatism, as illustrated by the reactions to the introduction of fast-food chains in the 1970s: France reacted to the expansion of American fast-food by adapting, to a certain extent, traditional national products to the fast-food formula [15]. Eating behaviors are influenced by numerous socio-demographic and behavioral variables, and French society is evolving just as fast as others. Increasing employment of women and general family dynamics, the later age of having a first child, immigration, and shorter lunch breaks all strongly influence eating habits [15–17].

In order to identify typical meal patterns, it is important to take into consideration a wide range of variables and to apply specific powerful statistical approaches, including clustering analysis. Dietary behaviors, with regard to their potential association with obesity, have to date largely been studied in children and adolescents [17]. In this study, we aimed to examine the so-called “French model” of eating behavior in adults in the Paris area, by identifying and characterizing distinct meal patterns.

## Methods

### Ethics statement

The SIRS cohort (a French acronym for “*Health*, *inequalities and social ruptures*”) study is a collaborative project between the French National Institute for Health and Medical Research (INSERM) and the National Centre for Scientific Research (CNRS), and received legal authorization from two French national authorities for non-biomedical research: the *Comité consultatif sur le traitement de l’information en matière de recherche dans le domaine de la santé* (CCTIRS) and the *Commission nationale de l’informatique et des libertés* (CNIL). The participants provide their verbal informed consent. Written consent was not necessary because this survey did not fall into the category of biomedical research (as defined by French law).

### Study population and design

Analyses were based on data from the 2010 wave of the SIRS cohort, a representative socio-epidemiological survey of the French-speaking adult (≥18) population conducted since 2005 in the Paris metropolitan area (population 6.5 million). The survey employed a stratified, 3-level random sampling procedure. In the first step, fifty census blocks with approximately 2000 inhabitants each were selected, over-representing the poorest neighborhoods. In the second step, sixty households were randomly selected from each surveyed census block. In the final step, one adult was chosen from each household by the birthday method. A questionnaire was administered face-to-face during home visits in 2005 and 2010, and detailed questions concerning meal structures and characteristics were introduced in 2010. In 2010, 47% of the original 2005 respondents were interviewed again face-to-face at home (2.6% had died, 1.8% were too sick to answer our questions, 2.7% were absent during the survey period, 13.9% had moved out of the 50 surveyed census blocks, 18.4% declined to participate, and 13.4% were lost to follow-up). The individuals who could not be reinterviewed were replaced by a random procedure similar to the one used in 2005, up to a final sample size of 60 adults by census block. The refusal rate in the newly contacted individuals was 29% (the same as in 2005). The methodology of the SIRS study has been further described elsewhere [18].

### Meal characteristics

Participants were asked about their most common meal-related habits in a typical week. In order to avoid any normative approach, meals were defined as “eating events” considered as meals eaten by the participants themselves [19]. Information concerning meals was collected without reference to a three-meal pattern, by referring to meals by their rank instead of their usual names (for further details, see [4]). We collected data on several meal characteristics, such as number, time, location (home, working place, restaurant), with whom the meal is usually taken (alone, with family members, with colleagues or friends) and activities associated with meals (television, radio, computer, reading, chatting). All those variables were categorized to be included in the clustering algorithm. Meal time was converted into 6 new variables displaying the occurrence of a meal during a time period (using as breaks: 12:30 a.m.; 5:30 a.m.; 10:30 a.m.; 2:30 p.m.; 6:30 p.m. and 10:30 p.m.). The intervals were chosen to correspond to commonly used meal times in France, but were large enough to adapt to diverse situations. Information on the other meal characteristics (location, other participants and activities) was included as proportions of daily meals displaying this characteristic, as it was important to avoid dependency on the number of meals.

### Clustering methods

In order to identify the different meal patterns, the partitioning around medoïds (PAM) algorithm with Manhattan distance was used as a reference analysis and applied to all the meal characteristic variables at once [20]. In order to determine the number of clusters, we used a resampling-based method and cluster-robustness approach called consensus clustering [21]. Sensitivity analyses were performed using three other clustering strategies: the same PAM algorithm with Euclidian distance, a *k*-means algorithm, and a hierarchical clustering algorithm. Analyses were conducted with *R* 2.15.3, using the *clusterCons* and *ggplot2* packages [22–24].

### Factors associated with meal patterns

Three different categories of factors were explored as being potentially associated with meal patterns. First, we considered social, demographic and economic characteristics such as gender, age (in five classes: 18–29; 30–44; 45–59; 60–74; 75 and over), level of education (in three classes: none or primary; secondary; higher degrees), occupation (five classes: employed; student; unemployed; retired; stay at home), household income per consumption unit (quartiles), living in an underprivileged neighborhood (according to the definition applied by the French government to target urban renewal programs and specific welfare policies), and origin (distinguishing between French, born to two French parents; French, born to at least one foreign parent; foreigner).

The second category depicted the social environment of the participant. It included the household type (four classes: single person household; couple with or without children; single-parent family; household with several unrelated individuals or families), the presence of a child under 16 years of age at home, and the feeling of loneliness.

The third category was made up of food-related characteristics, such as the occurrence of daily snacking (as defined by the participants themselves), dissatisfaction concerning food, level of involvement in meal-related decisions and in meal preparation, whether food quality was considered to be negatively affected by the participant’s lifestyle or financial issues. French national public health recommendations of eating five fruit or vegetables and three dairy products per day were also explored.

Associations between each of the socio-demographic and social environment variables and meal patterns were investigated using univariate multinomial regression models. Then, associations between each of the food-related variables and meal patterns were estimated with multinomial logistic regression models adjusted for socio-demographic and social environment characteristics. We reported unadjusted and adjusted odd ratios (OR) with their 95% confidence intervals and p-values. Analyses were conducted with *R* 3.0.3, with the *nnet* package [22,25].

### Prevalence estimates

To estimate prevalence in the reference population, some proportions presented in this article were weighted to account for the complex sample design (notably, the design effect associated with cluster sampling and the overrepresentation of poorer neighborhoods) and for the poststratification adjustment for age and gender according to the general population census data.

## Results

We retrieved data on meal characteristics for 2994 of the 3006 participants in the survey. We estimated that 66%, 24% and 8% of the Paris area adult population had three, two and four or more meals, respectively. In most cases, mealtimes matched the three timeslots for breakfast (5:30 a.m. to 10:25 a.m.), lunch (11:30 a.m. to 2:25 p.m.) and dinner (6:30 p.m. to 9:25 p.m.). When only two meals were declared, it was mainly because the participant skipped breakfast. For those who ate four or more meals, the additional meal was generally taken around 4:00 p.m. Further description of meal characteristics for the full sample are presented in table 1.

**Table 1:** Characteristics of meals in the whole sample and according to the five types of meal patterns.

		General		Type 1		Type 2		Type 3		Type 4		Type 5	
		n = 2994		n = 875 (29%)		n = 672 (22%)		n = 698 (23%)		n = 440 (15%)		n = 309 (10%)	
		*n*	*%*	*%*	p*	*%*	p*	*%*	p*	*%*	p*	*%*	p*
**Number of meals per day**		2994			<0.001		<0.001		<0.001		<0.001		<0.001
	1	109	3.6%	0%		0.3%		0.1%		18%		8.7%	
	2	680	22.7%	1.6%		5.4%		2.9%		78%		86.4%	
	3	1997	66.7%	89.9%		83.6%		88.5%		3.6%		4.5%	
	4	189	6.3%	7.3%		10%		7.9%		0.5%		0.3%	
	5	17	0.6%	1%		0.7%		0.4%		0%		0%	
	6	2	0.1%	0.1%		0%		0.1%		0%		0%	
**12:30 a.m. to 5:29 a.m.**		2994			0.553		0.367		0.073		0.662		0.296
	No	2925	97.7%	97.1%		96.9%		99%		97%		99%	
	Yes	69	2.3%	2.9%		3.1%		1%		3%		1%	
**5:30 a.m. to 10:29 a.m.**		2994			<0.001		<0.001		<0.001		<0.001		<0.001
	No	789	26.4%	4.2%		4.8%		2%		93.2%		95.8%	
	Yes	2205	73.6%	95.8%		95.2%		98%		6.8%		4.2%	
**10:30 a.m. to 2:29 p.m.**		2994			<0.001		0.809		0.004		<0.001		0.152
	No	248	8.3%	2.2%		7.6%		4.7%		25%		11.3%	
	Yes	2746	91.7%	97.8%		92.4%		95.3%		75%		88.7%	
**2:30 p.m. to 6:29 p.m.**		2994			0.342		0.003		0.81		0.935		0.422
	No	2609	87.1%	88.8%		82.6%		88%		87.7%		89.6%	
	Yes	385	12.9%	11.2%		17.4%		12%		12.3%		10.4%	
**6:30 p.m. to 10:29 p.m.**		2994			0.075		0.949		<0.001		<0.001		0.029
	No	340	11.4%	8.9%		11.8%		5.7%		21.1%		16.2%	
	Yes	2654	88.6%	91.1%		88.2%		94.3%		78.9%		83.8%	
**10:30 p.m. to 12:29 a.m.**		2994			0.994		0.925		0.015		0.047		0.58
	No	2796	93.4%	93.5%		93%		96.1%		90.5%		91.9%	
	Yes	198	6.6%	6.5%		7%		3.9%		9.5%		8.1%	
**At home**		2994			<0.001		<0.001		<0.001		<0.001		<0.001
	≤25%	58	1.9%	1.6%		0.7%		0%		0.2%		12.3%	
	26–50%	437	14.6%	17.6%		1.8%		0.9%		10.2%		71.2%	
	51–75%	845	28.2%	79.1%		10.4%		10.6%		0.5%		2.3%	
	>75%	1653	55.2%	1.7%		87%		88.5%		89.1%		14.2%	
**At work**		2994			<0.001		<0.001		<0.001		<0.001		<0.001
	≤25%	2014	67.3%	19.5%		95.7%		98.7%		95.7%		29.1%	
	26–50%	877	29.3%	72.2%		4%		1.3%		4.3%		61.5%	
	51–75%	78	2.6%	7.9%		0.3%		0%		0%		2.3%	
	>75%	25	0.8%	0.3%		0%		0%		0%		7.1%	
**In a restaurant**		2994			<0.001		<0.001		0.258		0.066		<0.001
	≤25%	2747	91.8%	88.2%		97.2%		91.3%		95.5%		85.8%	
	26–50%	229	7.6%	10.9%		2.5%		8.7%		4.5%		11.7%	
	51–75%	8	0.3%	0.8%		0.1%		0%		0%		0%	
	>75%	10	0.3%	0.1%		0.1%		0%		0%		2.6%	
**Eaten alone**		2994			<0.001		<0.001		<0.001		<0.001		<0.001
	≤25%	1127	37.6%	31.3%		1.5%		69.6%		32%		69.9%	
	26–50%	831	27.8%	40.3%		8.3%		29.1%		30.5%		27.5%	
	51–75%	419	14%	27.8%		24.7%		1.1%		0.5%		0%	
	>75%	617	20.6%	0.6%		65.5%		0.1%		37%		2.6%	
**Shared with family**		2994			<0.001		<0.001		<0.001		<0.001		<0.001
	≤25%	1089	36.4%	32.6%		76.9%		0.3%		41.6%		33%	
	26–50%	716	23.9%	38.9%		15.5%		1.6%		24.3%		49.8%	
	51–75%	563	18.8%	28.5%		7.1%		36.4%		1.1%		2.3%	
	>75%	626	20.9%	0.1%		0.4%		61.7%		33%		14.9%	
**Shared with colleagues/friends**		2994			<0.001		<0.001		<0.001		<0.001		<0.001
	≤25%	1869	62.6%	16.1%		92.7%		90.2%		95.9%		17.7%	
	26–50%	915	30.6%	69.7%		6%		8.2%		3.4%		63.6%	
	51–75%	131	4.4%	12.4%		0.9%		1.4%		0%		2.3%	
	>75%	73	2.4%	1.8%		0.4%		0.1%		0.7%		16.4%	
**Eaten in front of television**		2994			<0.001		<0.001		<0.001		<0.001		<0.001
	≤25%	1029	34.4%	43.2%		24.9%		39.4%		18.4%		41.4%	
	26–50%	851	28.4%	40.6%		18.8%		18.1%		18.2%		53.1%	
	51–75%	535	17.9%	15.3%		33.6%		24.2%		0.7%		1%	
	>75%	579	19.3%	0.9%		22.8%		18.3%		62.7%		4.5%	
**Eaten in front of computer**		2994			<0.001		0.108		<0.001		<0.001		0.464
	≤25%	2839	94.8%	92%		95.1%		98.6%		94.8%		93.9%	
	26–50%	121	4%	7.1%		3.1%		1.1%		3%		5.5%	
	51–75%	18	0.6%	0.9%		1.3%		0.1%		0%		0%	
	>75%	16	0.5%	0%		0.4%		0.1%		2.3%		0.6%	
**Eaten while reading**		2994			0.004		0.066		0.332		0.011		0.27
	≤25%	2779	92.8%	91.3%		91.5%		93.7%		94.3%		95.8%	
	26–50%	169	5.6%	7.2%		5.7%		5.7%		3.9%		3.6%	
	51–75%	24	0.8%	1.5%		1.2%		0.4%		0%		0%	
	>75%	22	0.7%	0%		1.6%		0.1%		1.8%		0.6%	
**Eaten while listening to the radio**		2994			<0.001		<0.001		0.163		<0.001		<0.001
	≤25%	2047	68.4%	63.7%		56.3%		64.3%		86.6%		91.3%	
	26–50%	656	21.9%	31%		23.8%		24.2%		8%		6.8%	
	51–75%	156	5.2%	4.9%		9.8%		6.7%		0%		0%	
	>75%	135	4.5%	0.5%		10.1%		4.7%		5.5%		1.9%	
**Eaten while chatting**		2994			<0.001		<0.001		<0.001		<0.001		<0.001
	≤25%	1016	33.9%	6.5%		80.2%		19.3%		63%		2.6%	
	26–50%	728	24.3%	37.3%		17.3%		14.8%		19.8%		31.2%	
	51–75%	580	19.4%	40.6%		1.8%		29.9%		0.2%		1%	
	>75%	669	22.4%	15.7%		0.7%		36%		17%		65.3%	

* p-value for comparison to whole sample (chi-square test)

### The five types of meal patterns

**Fig 1:**
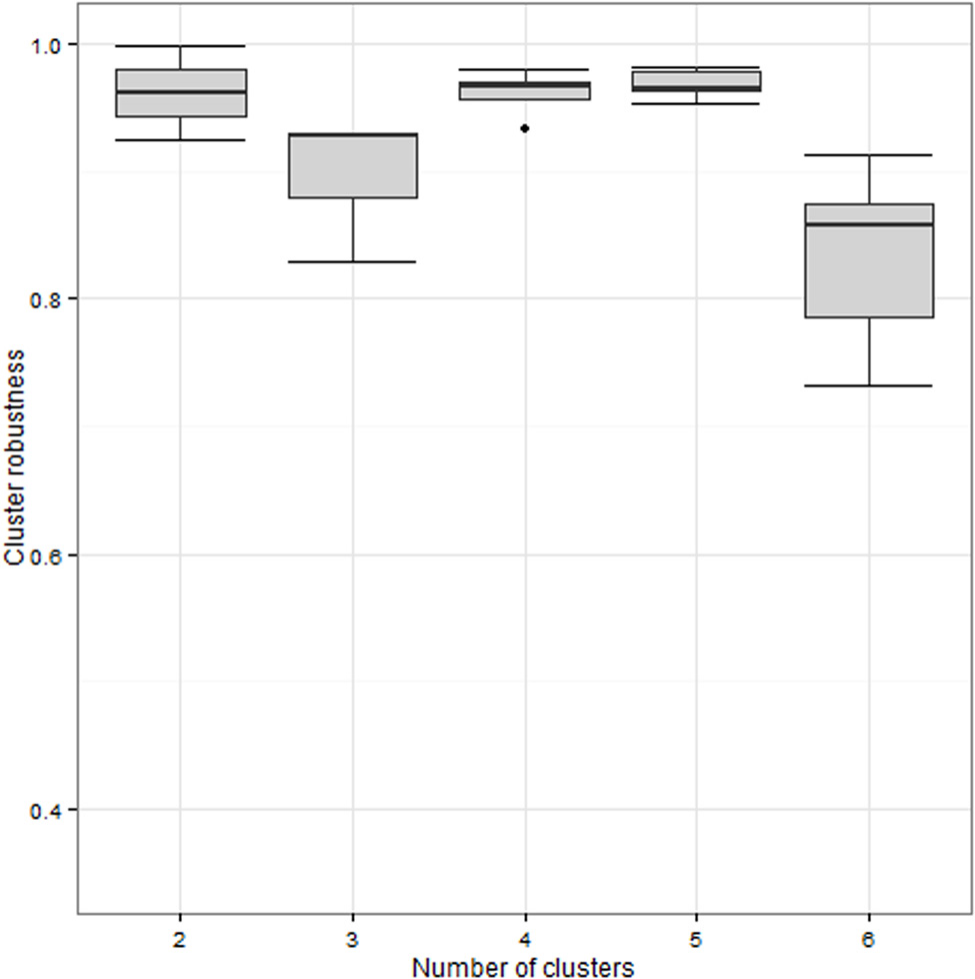
Cluster robustness according to the assumed number of clusters in the dataset (using the PAM algorithm with Manhattan distance). The cluster robustness evaluates the stability of groups while iterating the same clustering method with the same parameters, except for the assumed numbers of clusters in the dataset (from 2 to 6). The black line represents the median value, the bottom and top of the box represent the 1^st^ and 3^rd^ quartiles, and the ends of the whiskers represent minimum and maximum values. Highest median robustness with lowest dispersion was achieved considering 4- and 5-way classification. The examination of the relations between 4- and 5-way classifications revealed that while three groups were constantly retrieved in both cases, the fourth group in the 4-way classification split into two when the 5-way classification was applied (see Table 2).

Cluster robustness analysis indicated that the optimal number of clusters was five, even though the robustness of the division into four clusters was very close (Fig. 1). The examination of the relationship between the 4- and 5-way classifications revealed that while three groups were constantly retrieved in both classifications, the fourth group in the 4-way classification split into two when the 5-way classification was applied. Table 2 provides an example of this phenomenon for a single iteration. For this reason, we used the 5-way classification for all further analyses, while keeping in mind the higher-level proximity between groups 4 and 5.

**Table 2:** Cross-tabulation of 4- and 5-way classifications using the PAM algorithm with Manhattan distance.

		5-type division					
		*1*	*2*	*3*	*4*	*5*	*T*
**4-type division**	*1*	869	0	0	0	27	896
	*2*	0	670	0	1	0	671
	*3*	0	0	688	17	0	705
	*4*	6	2	10	**422**	**282**	722
	*T*	875	672	698	440	309	2994

*This table represents the number of individuals allocated to the different groups when classifying the study population into 4 or 5 groups*. *While the first 3 groups were constantly retrieved in both classifications*, *the fourth group in the 4-way classification split into two groups when the 5-way classification was applied*.

**Fig 2:**
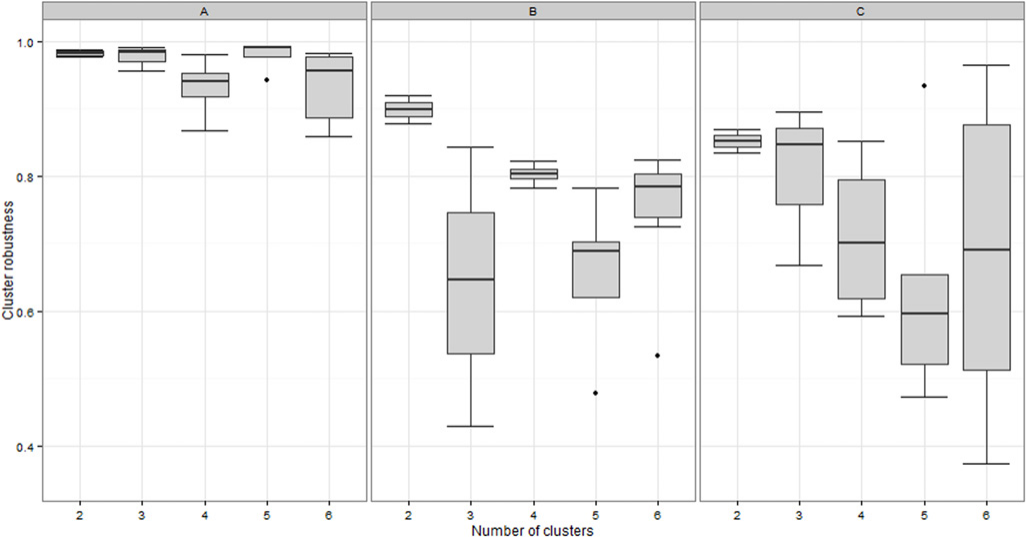
Sensitivity analyses: cluster robustness according to the assumed number of clusters in the dataset using (A) the PAM algorithm with Euclidian distance; (B) the *k*-means algorithm; and (C) a hierarchical clustering algorithm.

Cluster robustness using the PAM algorithm with a Euclidian distance also indicated that the optimal number of clusters was five (Fig. 2). The division was very similar to the one obtained using PAM with a Manhattan distance for groups 1–3, while clusters 4 and 5 were now mixed, and a new cluster including individuals eating constantly 4 meals per day appeared. With the *k*-means algorithm, the optimal number of clusters was four, and clusters were less clearly defined: clusters 4 and 5 were mixed again, and intermediate groups mixing individuals from clusters 1–2 and 1–3 appeared. Finally, the hierarchical clustering algorithm was far less specific in determining a robust optimal number of clusters, and classification in five clusters was thus not interpretable.

The five different types of meal pattern are described in table 1. The first type represented 29% of the sample (corresponding to an estimated prevalence of 33% of the adult, French-speaking population of the Paris metropolitan area). We have labelled this group “*3 meals*, *often outside*”. Participants commonly had three meals a day, within the usual timeslots. In comparison with the whole population, fewer meals were taken at home and with family members, though more meals were eaten at work or at the restaurant, with colleagues or friends. Activities undertaken during meals were less likely to be watching television, but rather chatting with other eaters.

In the second type, labelled “*3 meals*, *mostly alone at home*” (22% of the sample, corresponding to a prevalence of 17%), the three-meal pattern was also very frequent, though a higher proportion had a fourth meal (10%). Most people (87%) took their meals at home and in a large majority by themselves. A high proportion of these meals were eaten with television or radio, and very few were taken while chatting.

The third type of meal pattern was labelled “*3 meals*, *at home with family*” (23% of sample, corresponding to a prevalence of 24%). The three-meal pattern was generally adopted, and most of the meals were eaten at home, and shared with family members. Activities during meals were similar to the whole population.

The last two types both had a predominant 2-meal pattern, generally within the typical timeslots for lunch and supper, leading to the disappearance of the breakfast. The fourth type was named “*1 or 2 meals*, *mostly at home with television*” (15% of sample, corresponding to a prevalence of 13%), and was noteworthy by the large proportion of meals taken while watching television. Even though most meals were taken at home, often with family members, chatting during meals was relatively uncommon. A significant minority of 18% only ate one meal per day. Among these, less than 4% ate a meal in the morning, the median hour for their unique meal of the day being 7 p.m. (IQR: 4–8 p.m.). Eating one meal per day was not related to food insecurity nor to specific events that occurred during the last week (e.g. fasting, shifted work hours), although these participants more often declared having irregular eating habits (14% vs. 5%, p = 0.007).

The fifth type was named “*2 meals*, *often outside*” (10% of sample, corresponding to a prevalence of 12%). Most of the participants ate one or two meals per day (8.7% and 86.4%, respectively), with only approximately 5% eating a meal before 10:30 a.m. A very high proportion of meals were eaten away from home, at work or in a restaurant. Moreover, very few were eaten alone, as colleagues and friends frequently attended, and the main activity during those meals was chatting.

### Socio-demographic and environmental factors associated with meal patterns

Table 3 presents the results of the unadjusted multinomial regression. We chose to consider type 3 (“*3 meals*, *at home with family*”) as the reference group, since it was the closest to the traditional French meal pattern.

**Table 3:** Characteristics associated with meal patterns: univariate multinomial logistic regression, with type 3 (“3 meals, at home with family”) as reference.

		Type 1: *“3 meals*, *often outside”*	Type 2: *“3 meals*, *mostly alone at home”*	Type 4: *“1 or 2 meals*, *mostly at home with television”*	Type 5: *“2 meals*, *often outside”*	
		*OR [95% CI]*	*OR [95% CI]*	*OR [95% CI]*	*OR [95% CI]*	*p*
**Socio-demographic characteristics**						
**Gender**						<0.001
	Male	Ref	Ref	Ref	Ref	
	Female	1.04 [0.85–1.27]	1.49 [1.19–1.87]	0.75 [0.59–0.96]	0.61 [0.46–0.79]	
**Age (years)**						<0.001
	18–29	Ref	Ref	Ref	Ref	
	30–44	0.75 [0.53–1.06]	0.45 [0.29–0.7]	0.62 [0.41–0.93]	0.53 [0.35–0.79]	
	45–60	0.77 [0.54–1.09]	0.94 [0.62–1.42]	0.72 [0.48–1.08]	0.49 [0.32–0.75]	
	61–74	0.08 [0.05–0.13]	1.01 [0.68–1.51]	0.31 [0.2–0.48]	0.09 [0.05–0.16]	
	75+	0.02 [0.01–0.06]	2.17 [1.39–3.4]	0.36 [0.21–0.62]	0.02 [0.01–0.1]	
**Educational level**						<0.001
	College	Ref	Ref	Ref	Ref	
	High school	0.59 [0.48–0.74]	1.27 [1–1.59]	1.65 [1.27–2.13]	0.69 [0.52–0.92]	
	Primary/none	0.22 [0.14–0.33]	1.46 [1.06–2.01]	1.15 [0.78–1.69]	0.22 [0.12–0.4]	
**Occupation**						<0.001
	Employed	Ref	Ref	Ref	Ref	
	Student	1.14 [0.58–2.23]	2.64 [1.26–5.52]	1.55 [0.71–3.4]	1.46 [0.69–3.12]	
	Unemployed	0.08 [0.04–0.13]	0.92 [0.6–1.43]	1.21 [0.81–1.8]	0.18 [0.09–0.33]	
	Retired	0.03 [0.02–0.04]	1.53 [1.19–1.98]	0.36 [0.27–0.49]	0.06 [0.04–0.09]	
	At home/sick	0.01 [0–0.03]	0.45 [0.31–0.65]	0.35 [0.24–0.51]	0.03 [0.01–0.07]	
**Income per consumption unit (quartiles)**						<0.001
	€ 159–1600	Ref	Ref	Ref	Ref	
	€ 1601–2500	1.42 [1.03–1.96]	0.51 [0.37–0.69]	0.43 [0.31–0.61]	0.94 [0.63–1.41]	
	€ 2501–3900	1.19 [0.86–1.63]	0.33 [0.24–0.45]	0.31 [0.22–0.43]	0.78 [0.52–1.15]	
	€ 3901–40,000	1.24 [0.91–1.68]	0.16 [0.12–0.23]	0.16 [0.11–0.23]	0.6 [0.4–0.89]	
Living in an underprivileged neighborhood*						<0.001
	No	Ref	Ref	Ref	Ref	
	Yes	0.91 [0.71–1.15]	0.94 [0.73–1.21]	1.68 [1.29–2.18]	1.02 [0.75–1.4]	
**Parents nationality**						<0.001
	French, born to two French parents	Ref	Ref	Ref	Ref	
	French, born to one foreign parent	0.77 [0.6–0.99]	0.59 [0.45–0.78]	1.27 [0.95–1.7]	0.83 [0.59–1.16]	
	Foreigner	0.51 [0.38–0.7]	0.48 [0.34–0.67]	1.3 [0.94–1.79]	0.81 [0.55–1.19]	
**Social environment**						
**Family type**						<0.001
	Nuclear family	Ref	Ref	Ref	Ref	
	One person	52.86 [21.62–129.29]	325.13 [132.42–798.28]	80.27 [32.42–198.72]	53.76 [21.41–135]	
	Single parent	3.05 [2.14–4.34]	3.7 [2.39–5.73]	4.68 [3.15–6.95]	3.21 [2.06–5.01]	
	Multiple families	1.41 [0.82–2.44]	4.69 [2.69–8.16]	2.88 [1.61–5.14]	2.58 [1.38–4.84]	
**Children <16 at home**						<0.001
	No	Ref	Ref	Ref	Ref	
	Yes	0.97 [0.79–1.19]	0.19 [0.15–0.26]	0.67 [0.52–0.86]	0.99 [0.75–1.3]	
**Couple**						<0.001
	Live together	Ref	Ref	Ref	Ref	
	Do not live together	11.36 [5.41–23.82]	20.5 [9.43–44.54]	9.81 [4.35–22.1]	11.22 [4.93–25.57]	
	No couple	5.18 [3.95–6.79]	22.84 [17.02–30.65]	8.56 [6.34–11.58]	6.27 [4.51–8.7]	
**Solitude**						<0.001
	Does not feel lonely	Ref	Ref	Ref	Ref	
	Feels lonely	1.06 [0.76–1.48]	3.99 [2.95–5.39]	3.15 [2.26–4.39]	2.14 [1.46–3.14]	

*label applied by the French government to target urban renewal programs and specific welfare policies

Compared to the reference group, type 1 (“*3 meals*, *often outside*”) included significantly fewer people aged over 60, fewer inactive people (unemployed, retired or staying at home), fewer people with low or intermediate levels of education and fewer people of foreign origin. There was far more diversity in family types (more participants lived alone, in single-parent families or multiple families), but this is most likely explained by the remarkably high prevalence of couples (with or without children) in the reference group (89%).

Belonging to type 2 (“*3 meals*, *mostly alone at home*”) was particularly associated with living alone, but also with being female, of an advanced age, having a low or intermediate education level, having a low income and being retired. It was negatively associated with foreign origin. Type 2 was also related with being a student, but this probably reflects the low proportion of students in the reference group.

For participants belonging to type 4 (“*1 or 2 meals*, *mostly at home with television”*), the principal characteristics were being male, of a younger age, having a lower education level, a lower income, fewer children aged under 16 at home and notably living in an underprivileged neighborhood. We also observed that unemployed individuals and people of foreign origin were more likely to belong to this group (although the comparison with type 3 was not significant). Feeling of loneliness was important.

Type 5 (“*2 meals*, *often outside*”) was also associated with participants being male, of lower age and a higher sense of loneliness. But unlike type 4, type 5 was connected to higher educational levels.

Finally, the principal characteristics of the reference group (“*3 meals*, *at home with family*”) can be deduced from the multinomial regression. This pattern was strongly associated with participants who stayed at home, with a higher income, a nuclear family (couples with or without children) and an almost non-existent sense of loneliness. In this type, students or participants belonging to the 18–29 age group were infrequent.

### Food-related factors associated with meal patterns

Table 4 presents the results of a multinomial regression, with adjustment for each previously identified socio-demographic and environmental characteristic. Again, type 3 was chosen as the reference group. Daily snacking was reported significantly less frequently by individuals of type 1, and more frequently by individuals of types 4 and 5, who mostly followed a 2-meal per day pattern. Dissatisfaction concerning food was also more frequent in types 4 and 5. Involvement in meal-related decisions and meal preparation was remarkably frequent in type 2-in which most individuals lived alone. Food quality was considered to be affected by their lifestyle in types 1, 4 and 5, and by financial issues only in type 4. Finally, adherence to the 5-a-day fruit and vegetables guideline was infrequent in all 4 types, reflecting a frequent adherence in the reference group only, but was particularly rare in types 4 and 5 (as was adherence to the 3 dairy products per day guideline in both of these types).

**Table 4:** Food-related factors associated with meal patterns: univariate multinomial logistic regression with type 3 (“3 meals, at home with family”) as reference, with adjustment on socio-demographic and environmental characteristics.

		Type 1:*“3 meals*, *often outside”*	Type 2:*“3 meals*, *mostly alone at home”*	Type 4:*“1 or 2 meals*, *mostly at home with television”*	Type 5:*“2 meals*, *often outside”*	
		*OR [95% CI]*	*OR [95% CI]*	*OR [95% CI]*	*OR [95% CI]*	*p*
**Food**						
**Daily snacking**						<0.001
	No	Ref	Ref	Ref	Ref	
	Yes	0.72 [0.54–0.96]	0.81 [0.59–1.1]	2.51 [1.88–3.35]	2.12 [1.53–2.94]	
**Dissatisfaction concerning food**						<0.001
	No	Ref	Ref	Ref	Ref	
	Yes	1.08 [0.72–1.63]	1.25 [0.81–1.91]	2.03 [1.35–3.04]	2.34 [1.5–3.65]	
**Involvement in meal-related decisions**						0.016
	High	Ref	Ref	Ref	Ref	
	Low	1.07 [0.75–1.52]	0.54 [0.35–0.82]	0.8 [0.55–1.17]	1.08 [0.71–1.64]	
**Food quality affected by lifestyle**						<0.001
	No	Ref	Ref	Ref	Ref	
	Yes	1.48 [1.1–1.99]	1 [0.7–1.42]	1.97 [1.42–2.73]	2.06 [1.46–2.91]	
**Food quality affected by financial issues**						0.019
	No	Ref	Ref	Ref	Ref	
	Yes	0.96 [0.68–1.37]	0.92 [0.64–1.33]	1.49 [1.06–2.11]	0.94 [0.62–1.43]	
**Participation in food preparation**						<0.001
	More than once a week	Ref	Ref	Ref	Ref	
	Once a week	0.85 [0.5–1.43]	0.45 [0.23–0.88]	0.6 [0.34–1.08]	1.22 [0.68–2.17]	
	Once a month	1.42 [0.76–2.64]	0.39 [0.16–0.92]	0.56 [0.27–1.16]	1.25 [0.59–2.64]	
	Less or never	1.58 [1.02–2.44]	0.61 [0.37–1.01]	0.77 [0.49–1.22]	1.38 [0.83–2.3]	
**Eats 5 fruits and vegetables a day**						<0.001
	More than once a week	Ref	Ref	Ref	Ref	
	Once a week	1.19 [0.82–1.71]	1.35 [0.91–1.99]	1.94 [1.28–2.94]	1.26 [0.79–2.01]	
	Once a month	1.39 [0.97–1.98]	1.19 [0.82–1.74]	3.34 [2.31–4.83]	2.26 [1.5–3.43]	
	Less or never	1.62 [1.07–2.46]	1.41 [0.92–2.17]	5.07 [3.37–7.64]	2.96 [1.85–4.72]	
**Eats 3 dairy products a day**						<0.001
	More than once a week	Ref	Ref	Ref	Ref	
	Once a week	0.82 [0.56–1.22]	1.03 [0.68–1.56]	1.08 [0.71–1.66]	0.9 [0.54–1.5]	
	Once a month	1.39 [0.99–1.94]	0.93 [0.65–1.34]	1.54 [1.08–2.2]	2.11 [1.43–3.12]	
	Less or never	1.42 [0.95–2.13]	1.37 [0.91–2.06]	2.62 [1.76–3.88]	2.33 [1.46–3.7]	

## Discussion

Using robust clustering methods, we identified five different meal patterns, considering not only frequencies but also timeslots, locations, co-attendants and activities during meals. Meal frequencies have already been described in the SIRS survey [4]. We further explored the meal structures and characteristics in the same representative sample of the general adult, French-speaking population of the Paris metropolitan area. We were able to characterize socio-demographic and environmental factors associated with each pattern, and link them to several food-related behaviors, such as snacking, dissatisfaction concerning food, involvement in meal-related matters, and adherence to general public health policies concerning fruits and vegetables, and dairy products.

Most European countries share the 3-meal pattern with synchronized meal times [1]. Recent research aimed at understanding how daily meal patterns evolve showed that this pattern is still predominant. This is the case in the Nordic countries [14], in Belgium [26], in Italy [27], in Spain and to a lesser extent in the United Kingdom [28]. We showed that the majority (66%) of the adult population of the Paris metropolitan area still eats three meals per day. However, our cluster analysis allowed us to go further and investigate meal patterns in more detail. Our findings indicate that approximately a quarter of the adult population deviate from the traditional French three-meal pattern [29] by omitting breakfast (and sometimes another meal), a behavior that has been associated with low-quality diet and potential consequences for health [30]. Indeed, more than 95% of individuals belonging to types 4 or 5 only ate 1 or 2 meals per day, with a remarkable 18% of individuals of type 4 eating only one meal per day. This result is somewhat close to those from the Nordic survey, where an “unsynchronized” eating pattern was identified and characterized by a late start to the eating day, the displaced timing of eating and a smaller number of eating events. As mentioned by its authors, “most significantly, in all countries the probability of an unsynchronized eating rhythm among the unemployed is approximately double (and even more in Norway) than it is among those in working” [14].

Although our types 4 and 5 presented similar meal rhythms, both having a predominant two-meal pattern, multivariate analysis allowed us to distinguish two different specific meal patterns that would have otherwise been grouped in a single cluster. If types 4 and 5 both concerned rather young and single people, they corresponded to two quite different social profiles. On the one hand, type 4 concerned poorer, less educated, more frequently unemployed individuals who frequently lived in underprivileged neighborhoods and were of foreign origin. Therefore, their meal patterns may be related to different dimensions of social vulnerability. Notably, in the absence of any regular working hours, their day may be less structured and their meals desynchronized or skipped, as shown in some other research on poverty and food [31]. Type 5, on the other hand, can be interpreted better as an unsynchronized eating pattern representing a transitional life-phase that will pass when restrictions associated with family life and work start to exert their influence [14]. This interpretation is supported by the characteristics in type 5 subjects, who were young, active people with constraints on their time and a specific urban lifestyle. As for type 1, this emphasizes the strong influence of work and activity on eating patterns.

The existence of types 4 and 5 also raises the question of the subjective meaning of the meal and its highly social associations. Hence, for most people, a solitary meal is a undesirable situation, and may be not declared as a meal at all [32]. Sobal and Nelson have emphasized that “most research about the effects of eating alone on diet and nutrition focuses on the elderly, for whom living and eating alone are prevalent because of the high proportion of widows and widowers”. Conversely, our results showed that individuals in type 2-associated with advanced age, lower income, and living alone (maybe as a consequence of widowhood)—strongly adhered to the three-meal schedule (10% even ate 4 meals a day) and took their meals mostly at home, whereas eating alone and meal skipping principally concerned younger people. With regard to the literature about modernization of life styles and their effect on eating habits, the question is whether this phenomenon is transitional or whether it forecasts more sustainable changes in the way new generations eat.

Our results also highlight the issue of eating out, which is not well documented in France [33]. We showed that the three-meal pattern can take different forms according to the socio-demographic characteristics of eaters and the context in which meals are taken. Type 1 and to some extent type 5 offered new results about eating out that indicate that eating out is part of daily eating practices for a large part of the population of the Paris metropolitan area. This behavior can be attributed to work and time constraints, where interactions with friends and colleagues can replace those with family members, especially at lunchtime. However, it above all extends the previous definition of the French model of the three-meal pattern in which meals were typically taken at home. This pattern was formed during the 19th century on the bourgeois model of three meals a day, and gradually spread to society as a whole, becoming a cultural trait so widely shared by all classes that it became normative [29]. If the French still eat mostly at home [34], it is interesting to note that eating out is integrated into the three-meal pattern. In other words, contrary to what the more pessimistic literature would suggest, eating out is not necessarily synonymous of destructuration of eating habits nor of the obesity epidemic [35] but, in contrast, is actually smoothly integrated into the daily eating schedule.

Finally, our results also raise the issue of eating habits of immigrant populations. Paradoxically, individuals of foreign origin typically belonged either to type 4 (characterized by desynchronized eating rhythms and a majority of meals taken in front of the television) or to type 3 (the most consistent with the traditional French model, accounting for 24% of the Paris area population). Few studies have specifically addressed the question of immigrant socialization through food provisioning, cooking, commensality types, tastes and health outcomes in the French context. The available data has focused on food taken at home and has not investigated eating rhythms. However, it has been shown that eating away from home, particularly at school and factory canteens—which shape specific rules for meals (time slots, 3-course meals, dishes, etc.)—are important contexts for eating socialization, with respect to tastes and manners. Eating rhythms and places could thus be equally important as home cooking conditions. This result is even more surprising for immigrants coming from less educated backgrounds, which in France are less likely to follow the three-meal pattern [4]. Further research on this subject is needed to draw more precise conclusions.

This study has some limitations. Clearly, the population of the Paris area is not representative of the whole country in terms of social organization and living conditions. The data are based on declarations from participants that could be affected by a social desirability bias. However, we think that, in the context of the data collection of the SIRS cohort (where each participant was interviewed during at least 1-hour long sessions, and sometimes even more, on a large and varied set of questions about their living conditions, adverse experiences, and very intimate health and biographical events), such a bias might have been reduced since relationships of trust were built between participants and interviewers and food-related questions were not among the most intimate and sensitive ones addressed. Nonetheless, documentation of meal patterns in a typical week from self-report is clearly limited compared to other, more intensive methods of data collection such as the use of meal diaries over a given period of time. Moreover, only subjective and general perceptions of food quality have been studied. Data on actual nutrient intake would be necessary to further investigate food quality.

## Conclusion

Our findings offer new insights into the diversity of meal patterns in the Paris metropolitan area. This study used powerful methods to generate a taxonomy of eating patterns in the largest French metropolitan area. Even if the traditional French model is relatively conserved, we showed that a quarter of the population of the Paris area diverges from it. Social factors such as age, family type, income and occupation are strongly linked to meal patterns and eating behaviors. More specifically, people may divert from the traditional model in two ways that both involve skipping breakfast. In the first group, representing approximately 13% of the population, individuals typically eat one or two meals per day, often with an irregular schedule, at home and in front of the television. Individuals from this group frequently experience unemployment, lower income and living in underserved urban areas. Breakfast skipping, increased snacking, low adherence to dietary guidelines and frequent dissatisfaction about food quality suggest that there might also be health consequences to this meal pattern. In a second group, representing approximately 12% of the population, people also eat two meals or less per day, possibly with the same consequences on food quality. However, in this case, meals are often taken outside of the home, in social settings, and individuals from this group are typically active, integrated, young people, suggesting that this pattern might be an adaptation to a young, modern urban lifestyle. Only longitudinal research would reveal if this ‘generational’ pattern will change back towards a more traditional one, with ageing, family building or having children.
